# Neuroimaging outcomes in suspected papilledema

**DOI:** 10.1038/s41598-026-55133-4

**Published:** 2026-05-27

**Authors:** Theresia Knoche, Nehir Guelsoy, Charlotte Pietrock, Eberhard Siebert, Mirjam Rossel-Zemkouo, Zohreh Jami, Leon Alexander Danyel

**Affiliations:** 1 Department of Neurology, Charité – Universitätsmedizin Berlin – Campus Virchow Klinikum, Augustenburger Platz 1, 13353 Berlin, Germany; 2 Institute of Neuroradiology, Charité – Universitätsmedizin Berlin, Charitéplatz 1, 10117 Berlin, Germany; 3 Department of Ophthalmology, Charité – Universitätsmedizin Berlin – Campus Virchow Klinikum, Augustenburger Platz 1, 13353 Berlin, Germany

**Keywords:** papilledema, emergency imaging, neuroimaging, intracranial hypertension, cerebral mass lesions, optic disc edema, Diseases, Medical research, Neurology, Neuroscience

## Abstract

Papilledema due to elevated intracranial pressure may indicate life-threatening conditions such as intracranial mass lesions or cerebral sino-venous thrombosis. This bicentric retrospective cohort study evaluated neuroimaging outcomes and clinical predictors in adults presenting with suspected papilledema to emergency departments between 2009 and 2022. Diagnostic outcomes were categorized as confirmed papilledema (due to idiopathic intracranial hypertension, space-occupying lesions, hydrocephalus or secondary intracranial causes) or papilledema ruled out. Clinical features, visual acuity, and frequency of focal neurological deficits were compared between groups. Diagnostic yield and number needed to scan (NNS) for CT and MRI were calculated, and univariable logistic regression was performed to identify predictors of secondary intracranial causes. Among 225 patients (mean age 43.0 ± 16.8 years; 57% female), papilledema was confirmed in 124 (55%), including 44 (35.5%) with secondary intracranial causes. Papilledema was excluded in 73 patients (32.4%). Primary CT and MRI identified secondary pathology in 13.3% (NNS 7.5) and 16.7% (NNS 6) of patients, respectively. Diplopia, nausea/vomiting, and focal neurological deficits were associated with secondary causes but were not consistently present. Secondary intracranial pathology is common in suspected papilledema. Given the limited discriminatory value of clinical features, timely neuroimaging should be performed in the diagnostic evaluation.

**Supplementary Information:**

The online version contains supplementary material available at 10.1038/s41598-026-55133-4.

## Introduction

Papilledema, defined as optic disc edema secondary to elevated intracranial pressure (ICP), represents a critical neuro-ophthalmologic finding that may signal potentially life-threatening conditions such as intracranial mass lesions, hydrocephalus, or cerebral sino-venous thrombosis.

In clinical practice, true papilledema has to be distinguished from optic disc edema unrelated to intracranial pressure, as well as from pseudopapilledema, in which optic disc elevation mimics swelling but arises from benign conditions such as anatomical variants or optic disc drusen - calcified hyaline deposits within the optic nerve head that are often detected incidentally on imaging or during funduscopic examination^1^. As accurate identification of papilledema based on fundoscopic examination remains unreliable^1–3^, a substantial proportion of referrals for papilledema can ultimately be attributed to a misinterpretation of optic disc appearance^4,5^. Diagnostic uncertainty is further compounded by medicolegal concerns and limited access to specialized neuro-ophthalmologic care^6,7^. These factors likely contribute to the increasing number of emergency department (ED) evaluations for suspected papilledema in recent years^8,9^.

Patients with suspected papilledema are frequently referred to the ED, where neuroimaging is a cornerstone of initial assessment. Cranial CT often constitutes the first-line imaging modality due to its widespread availability and rapid image acquisition. However, CT entails exposure to ionizing radiation, which carries cumulative risks, particularly in younger patients and those requiring serial imaging^10^. While MRI is considered the preferred modality for comprehensive evaluation of suspected papilledema^11,12^, its application in the emergency care setting is constrained by limited availability.

In order to inform diagnostic pathways and optimize the use of neuroimaging in the ED setting, we evaluated neuroimaging outcomes in adult patients presenting with suspected papilledema. We compared demographic and clinical characteristics between patients with confirmed elevated ICP and those without, and aimed to identify predictors of papilledema resulting from secondary intracranial pathology.

## Results

Medical database inquiry identified 707 patient cases which were screened for study inclusion: Following the application of inclusion and exclusion criteria, the final study population comprised 225 patients (mean age 43 ± 16.8 years, 57% female). The patient selection process is detailed in *Supplementary Fig. **1*.

### Diagnostic outcome

**Fig. 1:**
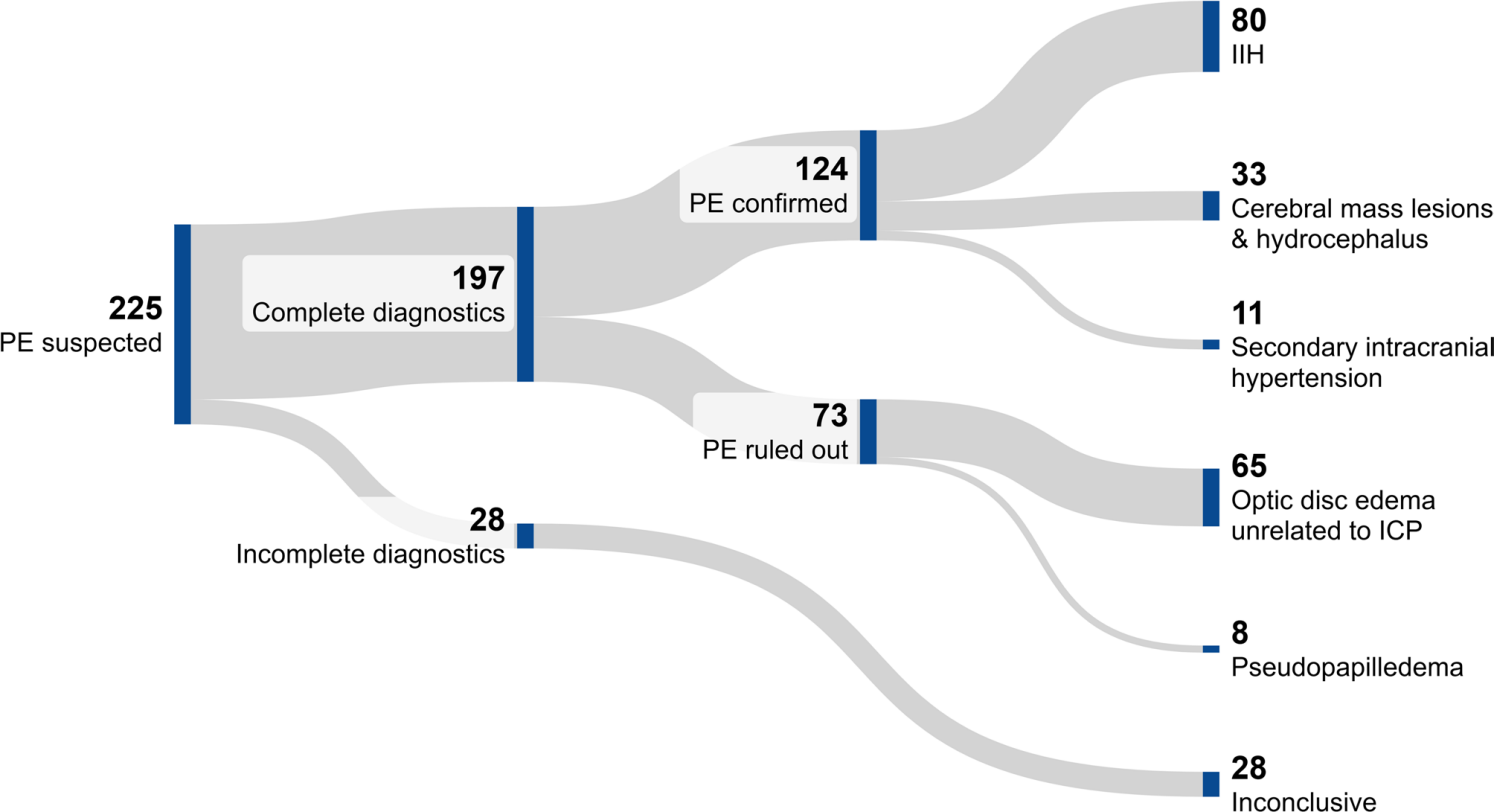
Sankey diagram illustrating the diagnostic outcome in patients with suspected papilledema. Abbreviations: ICP, intracranial pressure; IIH, idiopathic intracranial hypertension; PE, papilledema.

Papilledema was confirmed in 124 of 225 patients (Group 1: 55%; mean age 37 ± 13.8 years, 69% female). The most common underlying cause was idiopathic intracranial hypertension (IIH), accounting for 80 of 124 cases (Group 1a: 64.5%; mean age 35 ± 12.1 years, 80% female). Forty-four patients (Group 1b: 35.5%; mean age 41 ± 15.7 years, 48% female) had non-IIH-related papilledema, including 33 patients (26.6%) with cerebral mass lesions or hydrocephalus and 11 patients (8.9%) with secondary intracranial hypertension, most commonly due to cerebral sino-venous thrombosis (8 patients). Papilledema was excluded in 73 of 225 patients (Group 2: 32.4%; mean age 51 ± 17.4 years, 41% female). Among these, 65/73 patients (Group 2a: 89.0%) had optic disc edema unrelated to intracranial pressure (ICP), with etiologies including inflammatory optic neuropathies (*n* = 24), anterior ischemic optic neuropathy (*n* = 10), hypertensive optic neuropathy (*n* = 7), and other causes (*n* = 4). Notably, in 20 patients in whom papilledema was excluded, the cause of optic disc edema remained unidentified despite complete diagnostic workup, including lumbar puncture with CSF opening pressure measurement, neuro-ophthalmologic evaluation, and cerebral MRI. Pseudopapilledema was present in 8/73 patients (Group 2b: 11.0%; mean age 39 ± 15.2 years, 62.5% female), either related to anatomical variants (*n* = 5) or optic disc drusen (*n* = 3). Diagnostic evaluation was considered incomplete in 28 of 225 patients (12.4%) due to the absence of neuroimaging and/or cerebrospinal fluid (CSF) opening pressure measurements. Figure 1 illustrates the diagnostic outcome in patients with suspected papilledema. Table 1 details final diagnoses in patients with confirmed papilledema, optic disc edema unrelated to raised ICP and pseudopapilledema.

**Table 1:** Final diagnoses at discharge in patients with confirmed papilledema due to increased intracranial pressure (ICP), optic disc edema unrelated to ICP, and pseudopapilledema. In 20 patients without evidence of increased ICP, the etiology of optic disc edema remained undetermined despite a complete diagnostic workup (data not shown). Abbreviations: IIH, idiopathic intracranial hypertension.

Confirmed papilledema *n* = 124	Optic disc edema unrelated to ICP *n* = 45	Pseudopapilledema *n* = 8
IIH (*n* = 80)	Inflammatory causes (*n* = 24) Optic neuritis (*n* = 9) Papillitis (*n* = 8) Syphilitic optic neuropathy (*n* = 2) Posterior scleritis (*n* = 1) Chorioretinitis (*n* = 1) Intermediate uveitis (*n* = 1) Birdshot chorioretinopathy (*n* = 1) Paraneoplastic optic neuropathy (*n* = 1)	Anatomical variants (*n* = 5) Crowded disc (*n* = 2) Congenital anomaly (*n* = 1) Unspecified (*n* = 2)
Cerebral mass lesions (*n* = 20) Meningeoma (*n* = 12) Chronic subdural hematoma (*n* = 1) Glioblastoma (*n* = 2) Ganglioglioma (*n* = 1) Schwannoma (*n* = 1) Melanocytoma (*n* = 1) Anaplastic oligodendroglioma (*n* = 1) Metastatic non-small cell lung cancer (*n* = 1)	Ischemic optic neuropathy (*n* = 10)	Optic disc drusen (*n* = 3)
Hydrocephalus (*n* = 13) Obstructive hydrocephalus secondary to Neurocytoma (*n* = 1) Neuroectodermal tumor (*n* = 1) Pineal cyst (*n* = 1) Germinoma (*n* = 1) Colloid cyst (*n* = 1) Pilocytic astrocytoma (*n* = 1) Epidermoid cyst (*n* = 1) Non-obstructive hydrocephalus secondary to Leptomeningeal carcinomatosis (*n* = 3) Meningitis (*n* = 3)	Hypertensive optic neuropathy (*n* = 7)	
Secondary intracranial hypertension (*n* = 11) Sino-venous thrombosis (*n* = 8) Arteriovenous malformation (*n* = 1) Neuroborreliosis (*n* = 1) Neurosyphilis (*n* = 1)	Other causes (*n* = 4) Choroidal neoplasia (*n* = 2) POEMS syndrome (*n* = 1) Retinal detachment (*n* = 1)	

### Clinical presentation

**Fig. 2:**
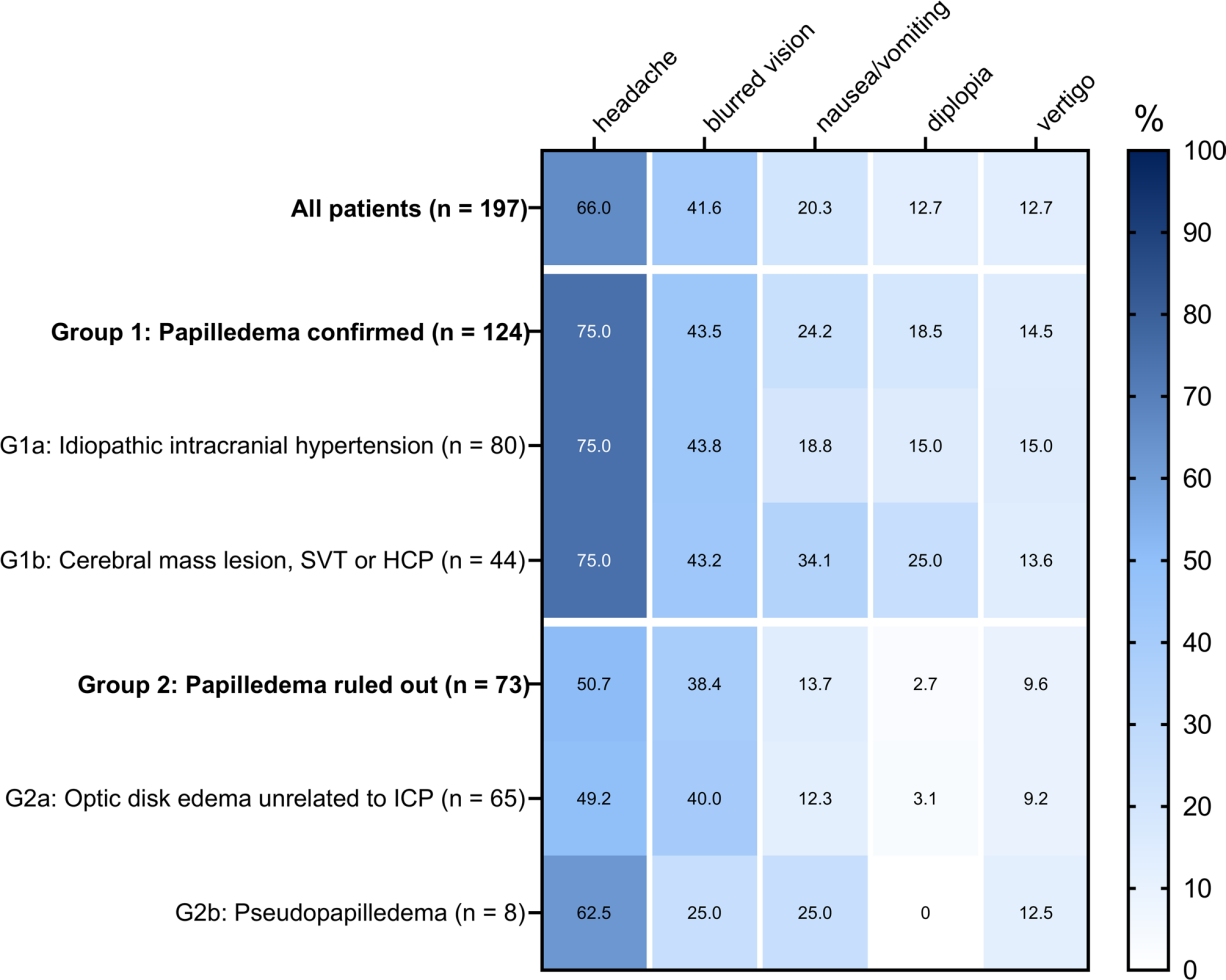
Heatmap illustrating the prevalence (total percentages) of common presenting symptoms in patients with suspected papilledema, stratified by diagnostic outcome (sub-)groups. Abbreviations: HCP, hydrocephalus; ICP, intracranial pressure; SIH, secondary intracranial hypertension.

Headache was the most frequently reported symptom, present in 66.0% of the overall cohort. It was particularly common in patients with confirmed papilledema (75.0%) but was also reported in those with optic disc edema unrelated to ICP (49.2%). Blurred vision was reported by 38.4–44.8% of patients across all subgroups, with the exception of those diagnosed with pseudopapilledema, in whom it was less frequent (25.0%). Vertigo and diplopia were less common, reported in 12.7% of all patients. However, diplopia was more common among patients with papilledema secondary to cerebral mass lesions, hydrocephalus, or secondary intracranial hypertension (25.0% of cases) and was rarely observed in patients with optic disc edema unrelated to elevated ICP (3.1% of cases). *Supplemental Table **1* provides a detailed description of presenting symptoms in patients with suspected papilledema, stratified by diagnostic outcome groups. Figure 2 illustrates the prevalence of presenting symptoms among patients evaluated for suspected papilledema.

Visual acuity (VA) was normal or mildly impaired (VA ≥ 0.3) in most patients (83.5%). Moderate visual impairment (VA < 0.3 to ≥ 0.1) was observed in 9.3% of cases, while severe impairment (VA < 0.1 to ≥ 0.05) and blindness (VA < 0.05) were reported in 3.6% and 3.1% of patients, respectively. The distribution of visual impairment was comparable across all subgroups (*Supplementary Table **2*).

New focal neurological deficits were observed in 22 of the 197 patients with suspected papilledema and complete diagnostic workup (11.2%). The most common deficit was abducens nerve palsy (*n* = 9), which occurred exclusively in patients with confirmed papilledema—specifically, in 3 patients with IIH and 6 with non-IIH-related papilledema. Anisocoria was documented in 6 patients overall and was consistently described as “mild” or “very mild,” with preserved pupillary light reflex in all cases (*n* = 1 with IIH, *n* = 2 with non-IIH-related papilledema and *n* = 3 with optic disc edema not attributable to increased ICP). Facial nerve palsy was identified in 3 patients, all of whom had underlying cerebral mass lesions. Additional focal findings included lateralized postural instability with up-beat nystagmus, homonymous quadrantanopia, severe impairment of smooth pursuit, and skew deviation - each observed in one patient respectively and all associated with either cerebral mass lesions or hydrocephalus. Hemiparesis was noted in two patients with IIH, both presenting with isolated mild pronator and rotator drift, the underlying cause remaining unclear. An overview of new focal neurological deficits across patient subgroups is provided in *Supplementary Table **3*.

In univariable analysis, diplopia (OR 3.3, 95% CI 1.2–9.2; *p* = 0.02), nausea and vomiting (OR 3.0, 95% CI 1.2–7.1; *p* = 0.02), and focal neurological deficits (OR 5.4, 95% CI 2.1–13.5; *p* < 0.001) were significantly associated with papilledema due to secondary intracranial pathology. No significant associations were found for headache and relative afferent pupillary defect (RAPD) (Table 2). Presence of blurred vision or vertigo, as well as visual acuity at presentation, did not differ significantly between diagnostic groups and were therefore not included in the regression analysis.

**Table 2:** Univariable logistic regression analysis of clinical predictors for papilledema due to secondary intracranial causes. Odds ratios (ORs) with 95% confidence intervals (95% CIs) are displayed. Diplopia, nausea and vomiting, and focal neurological deficits were significantly associated with papilledema caused by cerebral mass lesions, hydrocephalus, or secondary intracranial hypertension, whereas headache and relative afferent pupillary defect (RAPD) were not.

	OR (95% CI)	p-value
headache	1.9 (0.84 – 4.3)	0.12
diplopia	3.3 (1.2 – 9.2)	0.02
nausea & vomiting	3.0 (1.2 – 7.1)	0.02
focal neurological deficits	5.4 (2.1 – 13.5)	<0.001
RAPD	0.38 (0.12 – 1.2)	0.10

### Neuroimaging

**Fig. 3:**
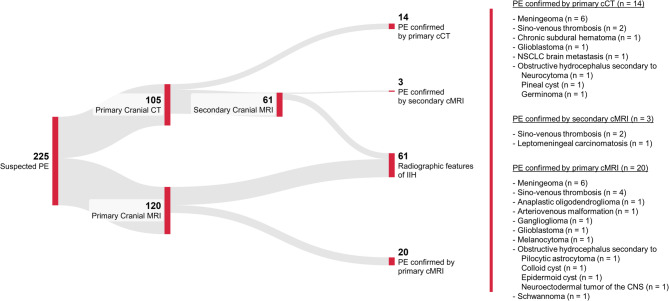
Sankey diagram illustrating the diagnostic yield of neuroimaging in 225 adult patients with suspected papilledema (PE). Initial imaging was performed with either cranial computed tomography (CT) or magnetic resonance imaging (MRI). Primary CT confirmed PE related to cerebral mass lesion, hydrocephalus or secondary intracranial hypertension in 14/105 (13.3%) patients (number needed to scan NNS: 7.5), while primary MRI identified 20/120 (16.7%) patients (NNS: 6). Notably, secondary MRI after primary CT identified PE unrelated to IIH in 3/61 (4.9%) patients (NNS: 20). Radiographic features of IIH were identified both by primary and secondary MRI in a total of 61 patients. Abbreviations: NSCLC, non-small-cell lung cancer.

Of the 225 patients included in the study, initial neuroimaging was performed using CT in 105 patients (47%) and MRI in 120 patients (53%), including MR venography in 99 patients (82.5%). Among patients with CT, angiography with venography was employed in a total of 33 patients, representing 31.4% of the subgroup. Among patients with unremarkable CT, 61 patients (67%, 61/91) subsequently underwent additional MRI (including MR venography in 51 patients, 83.6%). Figure 3 illustrates the diagnostic yield of neuroimaging in patients with suspected papilledema.

**Fig. 4:**
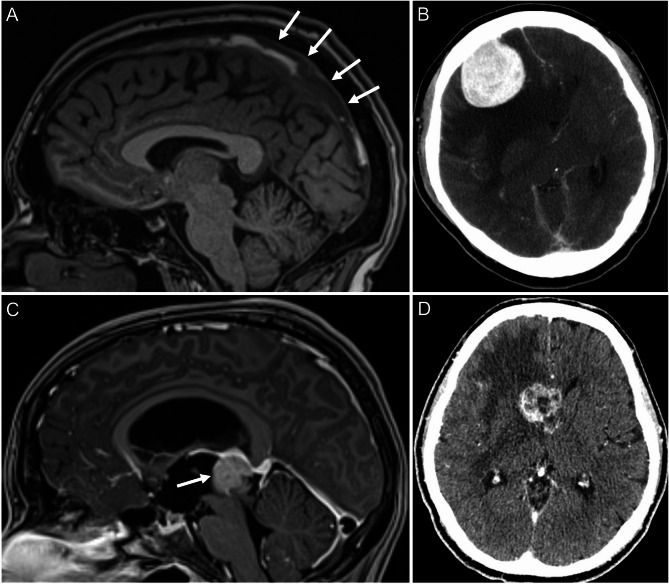
Neuroimaging examples in patients with confirmed papilledema unrelated to idiopathic intracranial hypertension. **A**: Sagittal T1-weighted MPRAGE MRI demonstrating extensive hyperintense thrombus in the superior sagittal sinus (white arrows) in a young patient with isolated abducens nerve palsy. **B**: Axial contrast-enhanced cranial CT scan of a patient with blurred vision and headache. No focal neurologic deficits were noted. A homogeneously enhancing, extra-axial mass is seen along the right frontal convexity with marked vasogenic edema and midline shift and subfalcine herniation. Histopathological analysis confirmed WHO grade I meningioma. **C**: Sagittal T1-weighted MPRAGE MRI in a patient presenting with blurred vision, diplopia, and headache. The image shows a large, lobulated germinoma in the pineal region (white arrow), occupying the posterior third ventricle and causing obstructive hydrocephalus. **D**: Axial post-contrast cranial CT depicting a centrally necrotic heterogeneous mass in the right head of the caudate nucleus, in the right frontal horn and adjacent corpus callosum with extensive perilesional edema. Histopathologic assessment confirmed NSCLC metastasis. Abbreviations: CT, computed tomography; MPRAGE, magnetization-prepared rapid acquisition gradient echo; MRI, magnetic resonance imaging; MIP, maximum intensity projection; NSCLC, non–small cell lung carcinoma; WHO, World Health Organization.

Primary CT confirmed the presence of papilledema by identifying intracranial mass lesions (*n* = 9), hydrocephalus (*n* = 3), or cerebral sino-venous thrombosis (*n* = 2) in 14 out of 105 patients (13.3%; number needed to scan, NNS = 7.5). In comparison, in patients who initially underwent MRI, non-IIH-related papilledema was confirmed in 20 of 120 patients (16.7%; NNS = 6), attributable to intracranial mass lesions (*n* = 11), hydrocephalus (*n* = 4), cerebral sino-venous thrombosis (*n* = 4) and to one case of arteriovenous malformation. Consecutive MRI after initially unremarkable CT examination confirmed non-IIH-related papilledema in 3 of 61 patients (4.9%, NNS = 20.3) attributable to cerebral sino-venous thrombosis (*n* = 2) and one case of leptomeningeal carcinomatosis. Examples of neuroimaging findings are presented in Fig. 4.

### Patients with imaging signs of IIH

MRI revealed imaging features suggestive of idiopathic intracranial hypertension without presence of cerebral mass lesion or cerebral sino-venous thrombosis in 61 of 181 patients (33.7%). Among these, IIH was diagnosed in 52 (85.2%) patients based on elevated CSF opening pressure and the exclusion of competing causes according to the diagnostic criteria by Friedman and colleagues^13^. Notably, 4 patients with imaging features of IIH and elevated CSF opening pressure had pleocytosis and were ultimately diagnosed with meningitis without confirmed pathogen (*n* = 3) and neurosyphilis (*n* = 1). Finally, 5 patients with subtle imaging features of IIH (slight widening of the optic nerve sheath) had normal CSF opening pressure. Here, optic disc edema was attributed to optic neuritis (*n* = 1), optic disc drusen (*n* = 1) or remained unclear in 3 patients. Frequencies of radiographic IIH signs are provided in Table 3.

**Table 3:** Frequencies of radiographic signs of IIH on MRI.

	partial empty sella	optic nerve sheath distension	transverse sinus stenosis	posterior globe flattening	optic nerve tortuosity
n	44	76	35	7	10
%	72.1	75.4	57.4	11.5	16.4

### Papilledema in patients with unremarkable neuroimaging

Neuroimaging in 127 of 225 patients (56.4%) revealed no evidence of cerebral space-occupying lesions, hydrocephalus, sino-venous thrombosis, or radiological signs indicative of intracranial hypertension. Within this subgroup, IIH was diagnosed in 28 patients (22.0%) based on elevated CSF opening pressure. Notably, one patient with elevated CSF opening pressure was subsequently diagnosed with neuroborreliosis. Two patients were diagnosed with leptomeningeal carcinomatosis.

## Discussion

In this cohort of adults presenting to the ED with suspected papilledema, approximately one in five patients demonstrated cerebral mass lesion, hydrocephalus, or cerebral sino-venous thrombosis on neuroimaging. Both CT and MRI exhibited high diagnostic yield (13.3%-16.7%) for detecting non–IIH related causes of papilledema, with a NNS of 6 for MRI and 7.5 for CT. The substantial prevalence of secondary intracranial pathology underscores the value of neuroimaging in patients referred for suspected papilledema.

In line with previous investigations, IIH was the most common cause of papilledema, accounting for 64.5% of cases^14^. However, more than one third of these patients did not exhibit radiographic features of IIH on MR imaging. This observation is in line with previous investigations, which reported limited sensitivity for MRI in IIH^15,16^. Importantly, imaging features suggestive of IIH may be present in other diseases associated with increased intracranial pressure or may represent incidental findings in healthy patients^17^. As such, the presence of IIH imaging features may contribute supportive evidence when integrated into the clinical and diagnostic context^13^.

All patients in our cohort were referred by ophthalmologists, either from outpatient care or through the ophthalmology departments of the participating centers. Still, papilledema due to increased ICP was excluded in one-third of patients. These patients were either found to have optic disc edema unrelated to ICP or pseudopapilledema. Most common causes of optic disc edema included inflammatory, ischemic, or hypertensive optic neuropathies. However, the broad spectrum of neuro-ophthalmological disorders observed highlights the complexity of differential diagnosis in this cohort and underscores the need for a comprehensive neurological and neuro-ophthalmological assessment. Our findings are in line with published data, showing that up to one-third of patients referred for suspected papilledema in acute care settings are ultimately diagnosed with optic disc edema unrelated to ICP or pseudopapilledema^4,8^. Notably, overdiagnosis of IIH has been largely attributed to a misinterpretation of optic nerve appearance^5^. Our analysis identified focal neurological deficits, diplopia, and nausea/vomiting as being associated with papilledema due to secondary intracranial pathology. The majority of patients with secondary intracranial pathology did not exhibit any focal neurological deficits. Furthermore, abducens nerve palsy - the most frequently observed deficit - was also present in a subset of patients with IIH and diplopia was a frequently reported symptom across both groups. Interestingly, anisocoria was documented in patients with both papilledema and optic disc edema unrelated to elevated ICP and was consistently described as mild, with preserved pupillary light reflex. We hypothesize that, in the context of suspected papilledema, physiologic anisocoria—which is present in approximately 15–20% of the general population—may be prone to misinterpretation as a pathological finding^18^. Although headache was highly prevalent in our cohort, it did not discriminate between etiologies. Similarly, neither blurred vision and vertigo, nor objective measures such as visual acuity, did differentiate between groups. Taken together, these findings underscore that while certain clinical features may raise suspicion for papilledema to intracranial pathology, symptom-based stratification alone is insufficient to guide neuroimaging decisions in suspected papilledema.

Given the substantial prevalence of intracranial pathology, the nonspecific nature of presenting symptoms, and the low frequency of focal neurological deficits, a strategy of routine cerebral imaging, rather than reliance on prior clinical risk stratification, seems justified. In our cohort of 225 patients, only one patient required urgent surgical intervention (evacuation of subdural hematoma). Although expert recommendations advocate urgent neuroimaging in all patients with suspected papilledema, formal guideline recommendations regarding the exact timing of imaging are lacking. In clinically stable patients without acute neurological deterioration, MRI within 24–48 h may represent a safe and practical approach. This circumvents the limitations of emergency MRI availability in acute care settings and avoids unnecessary radiation exposure from nonessential CT examinations^10,19–22^. MR venography should be included in the imaging protocol to exclude cerebral venous sinus thrombosis or detect transverse sinus stenosis related to IIH. The American Heart Association recommends contrast-enhanced MR venography for improved thrombus detection—particularly in small veins—through direct visualization of luminal filling, while reducing flow-related artifacts^23^.

Our study has several limitations inherent to its retrospective design: Patient selection was based on a medical database query using ICD-10 code H47.1 (“papilledema”) and may therefore have failed to identify patients in cases of miscoding or incomplete documentation. Importantly, the term papilledema, which denotes optic disc edema due to elevated ICP, is commonly misapplied in clinical practice and the medical literature^24^. As such, we individually reviewed patient data to only include patients with optic disc edema referred on the clinical suspicion of increased ICP. In addition, patients with previously diagnosed papilledema or conditions associated with elevated ICP were excluded. As symptom data such as headache, diplopia, or blurred vision were patient-reported and not collected by standardized interview, their apparent frequency could be affected by underreporting or inconsistent documentation. Moreover, the etiology of optic disc edema remained unclear in 12% of cases due to incomplete workup, attributed to missing CSF opening pressure measurements or neuro-ophthalmological examinations. With regards to neuroimaging, heterogeneous imaging protocols were used both for CT and MRI, with varying use of contrast agent and inclusion of venography relevant for identification of sino-venous thrombosis, which likely affected diagnostic outcome in our study. Imaging features of IIH were not assessed using a standardized protocol. Instead, the reported frequencies reflect the findings documented in the original radiology reports generated at the time of patient management, which may have influenced their observed prevalence. Furthermore, not all commonly described imaging features of IIH were systematically evaluated, e.g. the prevalence of meningoceles as described by Bialer et al.^25^.

Finally, our study reflects the diagnostic practices of two tertiary care centers, which limits generalizability to other healthcare settings. Despite these limitations, our study has notable strengths. Including 225 adult patients, it represents one of the largest cohorts systematically evaluated for suspected papilledema in the acute care setting, providing insights into patient demographics, symptomatology, neuroimaging utility, and the overall diagnostic spectrum.

To conclude, in adults presenting to the ED with suspected papilledema, secondary intracranial pathology such as cerebral mass lesion, hydrocephalus, or cerebral venous sinus obstruction is common. Given the poor discriminatory value of presenting symptoms and focal neurologic deficits, cerebral imaging should be performed routinely in all patients, rather than selected on the basis of clinical risk stratification.

## Methods

This is a bicentric retrospective observational study investigating the clinical presentation, neuroimaging and diagnostic outcome of patients presenting with suspected papilledema in emergency care. All patients were evaluated at one of two tertiary care hospitals affiliated with the Charité – Universitätsmedizin Berlin. Ethical approval was obtained from the institutional review board (ethics committee of Charité – Universitätsmedizin Berlin, application number: EA1/169/23). The requirement to obtain participant consent was waived due to the retrospective nature of the study. All methods were performed in accordance with relevant guidelines and regulations and in compliance with the Declaration of Helsinki. The study was designed and reported in compliance with the Strengthening the Reporting of Observational Studies in Epidemiology (STROBE) Statement^26^.

### Patient selection

A medical database inquiry identified consecutive patients treated under the definite or suspected diagnosis of papilledema (ICD-10 code: H47.1) between January 2009 and November 2022. Patients were eligible for inclusion if they met the following criteria: (1) age ≥ 18 years; (2) availability of an ophthalmologic assessment at the index visit; and (3) referral for further evaluation based on clinical suspicion of papilledema. Patients with a prior diagnosis of papilledema or any known condition associated with elevated ICP were excluded.

### Data collection

Data were extracted from electronically archived records, radiology reports, and neuro-ophthalmology consultation notes. Presenting symptoms were recorded as the presence or absence of headache, blurred vision, vertigo, diplopia, relative afferent pupillary defect (RAPD) and nausea/vomiting. Neurological examination findings were reviewed for the presence of new focal neurological deficits suggestive of raised ICP (i.e. lateralized paresis or sensory loss, cranial nerve palsy, central oculomotor disorder, dysarthria, aphasia, ataxia and/or homonymous visual field defects). Impairment of visual acuity was categorized according to the World Health Organization International Statistical Classification of Diseases and Related Health Problems (10th revision, 2016). Neuroimaging data included the primary imaging modality performed during emergency care, as well as secondary cranial MRI in patients who initially underwent cranial CT. Imaging findings were documented, including the presence of radiological signs suggestive of idiopathic intracranial hypertension (IIH)^27^. Results of diagnostic lumbar puncture, CSF opening pressure measurements, were recorded. Findings from neuro-ophthalmology consultations were documented, including key examination results and the final ophthalmologic diagnosis. Based on a comprehensive review of all available clinical and paraclinical data, patients were categorized into three groups:

*Group 1 – Papilledema confirmed*: Papilledema was confirmed in patients presenting with optic disc edema attributable to either (i) a cerebral mass lesion and/or hydrocephalus identified on neuroimaging, or (ii) elevated CSF opening pressure (≥ 25 cmH₂O) on lumbar puncture. This group was further subclassified into:

*Group 1a*: Papilledema due to IIH, defined according to the modified Friedman criteria^13^.

*Group 1b*: Papilledema secondary to space-occupying lesions, hydrocephalus, or secondary intracranial hypertension.

*Group 2 – Papilledema ruled out*: Papilledema was ruled out in patients with unremarkable neuroimaging and normal CSF opening pressure. This group included:

*Group 2a*: Patients with optic disc edema unrelated to elevated ICP.

*Group 2b*: Patients with pseudopapilledema.

*Group 3 – Inconclusive*: Patients for whom the diagnostic workup was incomplete, and a definitive diagnosis could not be established. These patients were excluded from secondary analyses related to symptomatology, visual impairment and focal neurological deficit.

### Statistical analysis

Statistical analyses were performed using IBM SPSS Statistics (Version 30.0. Armonk, NY: IBM Corp.). GraphPad Prism (Version 8.0.0 for Windows, San Diego, CA, USA) was used for graphic illustration. Categorical variables are expressed as frequencies and percentages. Frequencies of common presenting symptoms, grade of visual impairment and focal neurological deficits are presented as total percentages; corresponding valid percentages and missing data are provided in the supplementary material. The diagnostic yield of CT and MRI for identifying papilledema attributable to cerebral mass lesions, hydrocephalus, or secondary intracranial hypertension was calculated. In addition, the number needed to scan (NNS) was derived as the reciprocal of the diagnostic yield.

Univariable binary logistic regression analysis was employed to identify clinical predictors associated with papilledema due to secondary intracranial causes. The regression analysis was designed to identify patients with papilledema resulting from cerebral mass lesions, hydrocephalus, or secondary intracranial hypertension (Group 1b) within the remaining cohort, which comprised patients with IIH-related papilledema (Group 1a) and those with optic disc edema unrelated to ICP/pseudopapilledema (Group 2a & Group 2b). Variables were selected for univariable regression analysis based on differences observed in descriptive group comparisons and their presumed clinical relevance as potential predictors of secondary intracranial pathology. The variables included headache, diplopia, nausea and vomiting, vertigo, RAPD, and focal neurological deficits. Missing data was handled by pairwise deletion and reporting of valid percentages. Results are reported as odds ratios (ORs) with confidence intervals (95% CIs). The level of significance was set at a two-sided p-value < 0.05.

## Supplementary Information

Below is the link to the electronic supplementary material.

Supplementary Material 1

## Data Availability

The datasets generated for the current study are not publicly available but are available from the corresponding author on reasonable request.
