# Supplementary material for: Neuroimaging outcomes in suspected papilledema
Source: Sci Rep. 2026 May 27;16:16372. doi: 10.1038/s41598-026-55133-4 (PMC13212560; doi:10.1038/s41598-026-55133-4)
Supplement: Supplementary file 1 — Supplementary Material 1 [file 41598_2026_55133_MOESM1_ESM.docx]

**Supplemental Material**


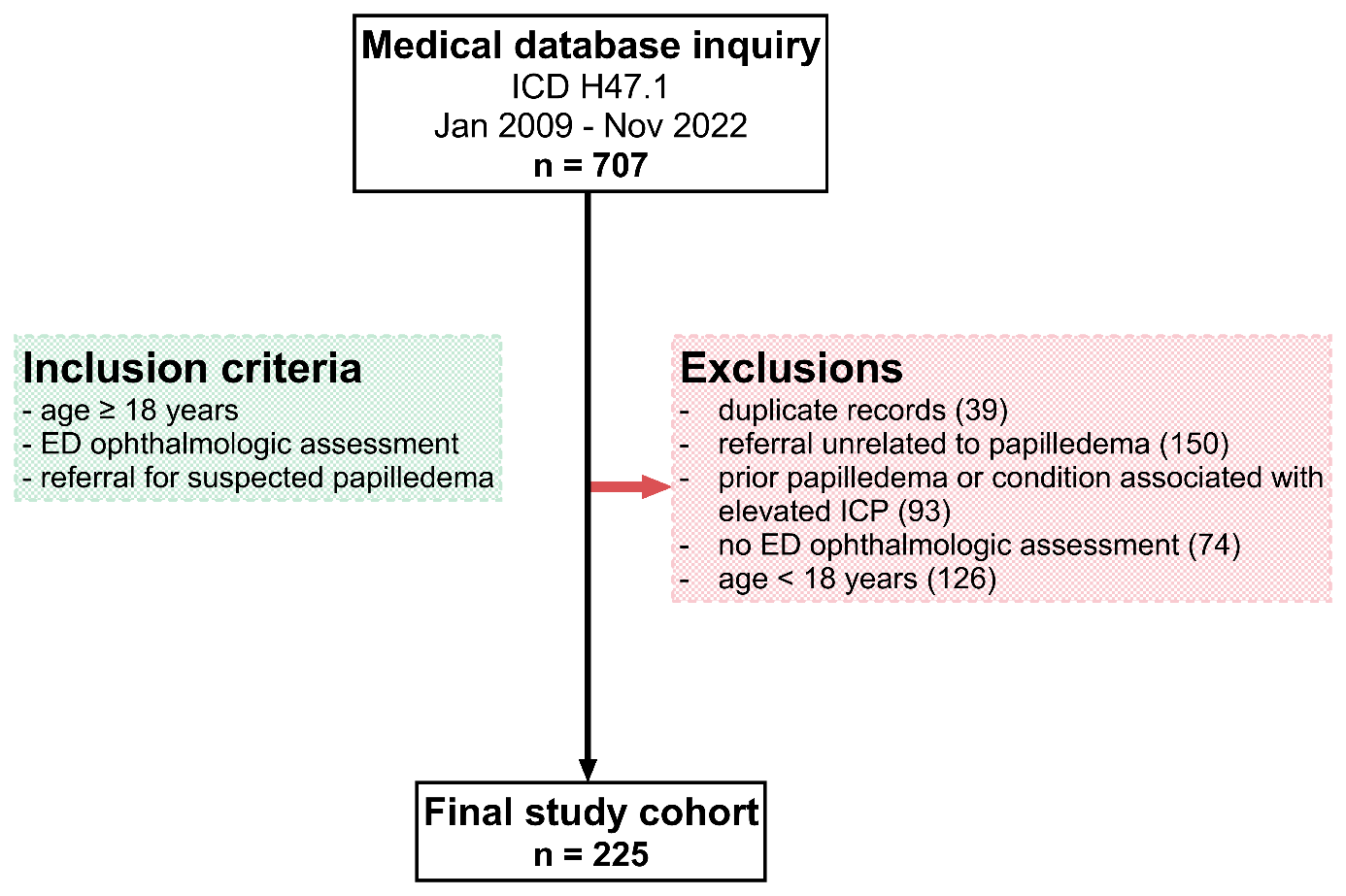


**Supplementary Figure 1: Flow diagram of the patient selection process. Abbreviations: ED, emergency department; ICD, International Classification of Diseases; ICP, intracranial pressure**

**Supplementary Table 1: Frequencies (absolute numbers and valid percentages) of common presenting symptoms in patients with suspected papilledema, stratified by outcome groups.** Abbreviations: ICP, intracranial pressure; IIH, idiopathic intracranial hypertension; SIH, secondary intracranial hypertension.

|  | **Blurred vision** | **Diplopia** | **Headache** | **Nausea/ vomiting** | **Vertigo** |
| --- | --- | --- | --- | --- | --- |
| **Patients w/ suspected papilledema (n=197)** | 82/87  (94.3%) | 25/88  (28.4%) | 130/189  (68.7%) | 40/117  (34.2%) | 25/40  (62.5%) |
| **Group 1**  – papilledema confirmed (n = 124) | 54/59  (91.5%) | 23/64  (35.9%) | 93/122  (76.2%) | 30/83  (36.1%) | 18/29  (62.1%) |
| **Group 1a**  – papilledema in IIH (n= 80) | 35/40  (87.5%) | 12/41  (29.3%) | 60/80  (75%) | 15/55  (27.3%) | 12/22  (54.5%) |
| **Group 1b**  – papilledema due to cerebral lesions, SIH or hydrocephalus (n =44) | 19/19  (100%) | 11/23  (47.8%) | 33/42  (78.6%) | 15/28  (53.6%) | 6/7  (85.7%) |
| **Group 2**  – papilledema ruled out (n = 73) | 28/28  (100%) | 2/24  (8.3%) | 37/67  (55.2%) | 10/34  (29.4%) | 7/11  (63.6%) |
| **Group 2a**  – optic disc edema unrelated to ICP (n = 65) | 26/26  (100%) | 2/21  (9.5%) | 32/60  (53.3%) | 8/29  (27.6%) | 6/9  (66.7%) |
| **Group 2b**  – pseudopapilledema (n= 8) | 2/2  (100%) | 0/3  (0%) | 5/7  (71.4%) | 2/5  (40%) | 1/2  (50%) |

**Supplementary Table 2: Distribution of visual impairment (absolute numbers and valid percentages) according to the World Health Organization International Statistical Classification of Diseases and Related Health Problems (10th revision, 2016).** Abbreviations: ICP, intracranial pressure; IIH, idiopathic intracranial hypertension; SIH, secondary intracranial hypertension; VA, visual acuity; VI, visual impairment.

|  | **Mild or no VI**  **VA ≥ 0.3** | **Moderate VI**  **VA < 0.3/≥0.1** | **Severe VI**  **VA < 0.1/≥0.05** | **Blindness**  **VA < 0.05** |
| --- | --- | --- | --- | --- |
| **Patients w/ suspected papilledema (n=197)** | 162/194  (83.5%) | 18/194  (9.3%) | 7/194  (3.6%) | 6/194  (3.1%) |
| **Group 1**  – papilledema confirmed (n = 124) | 108/122  (88.5%) | 7/122  (5.7%) | 3/122  (2.5%) | 3/122  (2.5%) |
| **Group 1a**  – papilledema in IIH (n= 80) | 74/79  (93.7%) | 3/79  (3.8%) | 1/79  (1.3%) | 0/79  (0%) |
| **Group 1b**  – papilledema due to cerebral lesions, SIH or hydrocephalus (n =44) | 34/43  (79.1%) | 4/43  (9.3%) | 2/43  (4.7%) | 3/43  (7%) |
| **Group 2**  – papilledema ruled out (n = 73) | 54/72  (75.0%) | 11/72  (15.3%) | 4/72  (5.6%) | 3/72  (4.2%) |
| **Group 2a**  – optic disc edema unrelated to ICP (n = 65) | 46/ 64  (71.9%) | 11/64  (17.2%) | 4/64  (6.3%) | 3/64  (4.7%) |
| **Group 2b**  – pseudopapilledema (n= 8) | 8/8  (100%) | 0/8  (0%) | 0/8  (0%) | 0/8  (0%) |

**Supplementary Table 3: Distribution of focal neurological deficits (FND) among patients with suspected papilledema (absolute numbers and valid percentages).** *Other FND included homonymous quadrantopia, impaired smooth pursuit, skew deviation, upbeat nystagmus and lateralized postural instability. Abbreviations: ICP, intracranial pressure; IIH, idiopathic intracranial hypertension; SIH, secondary intracranial hypertension.

|  | Any FND | Abducens nerve palsy | Anisocoria | Facial nerve palsy | Hearing loss | Hemi-paresis | Hemi-sensory loss | Trochlear nerve palsy | Other FND* |
| --- | --- | --- | --- | --- | --- | --- | --- | --- | --- |
| **Patients w/ suspected papilledema (n=197)** | 31/197  (15.7%) | 9/197  (4.6%) | 6/197  (3.0%) | 3/197  (1.5%) | 2/197  (1.0%) | 2/197  (1.0%) | 3/197  (1.5%) | 1/197  (0.5%) | 5/197  (2.5%) |
| **Group 1**  – papilledema confirmed (n = 124) | 27/124  (21.8%) | 9/124  (7.3%) | 3/124  (2.4%) | 3/124  (2.4%) | 2/124  (1.6%) | 2/124  (1.6%) | 3/124  (2.4%) | 0/124  (0.0%) | 5/124  (4.0%) |
| **Group 1a**  – papilledema in IIH (n= 80) | 9/80  (11.3%) | 3/80  (3.8%) | 1/80  (1.3%) | 0/80  (0.0%) | 1/80  (1.3%) | 2/80  (2.5%) | 2/80  (2.5%) | 0/80  (0.0%) | 0/80  (0.0%) |
| **Group 1b**  – papilledema due to cerebral lesions, SIH or hydrocephalus (n =44) | 18/44  (40.9%) | 6/44  (13.6%) | 2/44  (4.5%) | 3/44  (6.8%) | 1/44  (2.3%) | 0/44  (0.0%) | 1/44  (2.3%) | 0/44  (0.0%) | 5/44  (11.4%) |
| **Group 2**  – papilledema ruled out (n = 73) | 4/73  (5.5%) | 0/73  (0.0%) | 3/73  (4.1%) | 0/73  (0.0%) | 0/73  (0.0%) | 0/73  (0.0%) | 0/73  (0.0%) | 1/73  (1.4%) | 0/73  (0.0%) |
| **Group 2a**  – optic disc edema unrelated to ICP (n = 65) | 4/65  (6.2%) | 0/65  (0.0%) | 3/65  (4.6%) | 0/65  (0.0%) | 0/65  (0.0%) | 0/65  (0.0%) | 0/65  (0.0%) | 1/65  (1.5%) | 0/65  (0.0%) |
| **Group 2b**  – pseudopapilledema (n= 8) | 0/8  (0.0%) | 0/8  (0.0%) | 0/8  (0.0%) | 0/8  (0.0%) | 0/8  (0.0%) | 0/8  (0.0%) | 0/8  (0.0%) | 0/8  (0.0%) | 0/8  (0.0%) |
